# Reactive gliosis mimicking tumor recurrence – a case series documenting MRI abnormalities and neuropathological correlates 

**DOI:** 10.5414/NP301084

**Published:** 2018-02-09

**Authors:** Hugh Kearney, Jane Cryan, Alan Beausang, Seamus Looby, Francesca M. Brett

**Affiliations:** 1 Department of Neuropathology and; 2 Department of Neuroradiology, Beaumont Hospital, Dublin, Ireland

**Keywords:** gliosis, hemostatic agent, textiloma, gossypiboma

## Abstract

Abstract. The aim of this study is to identify, in our center, all cases of foreign-body reactions to hemostatic agents or other prostheses resulting in a radiological suspicion of tumor recurrence. We interrogated our internal database to identify all such cases and systematically evaluated the MRI brain scans of patients: (i) at the time of initial tumor diagnosis, (ii) postoperatively, (iii) and at the time of suspected tumor recurrence. In addition, we reviewed each patient’s operative notes and reviewed the histology of all cases following a second surgical intervention. In total, we identified 8 patients, 7 of whom had a WHO grade II glioma at initial surgery. We did not identify any distinguishing radiological abnormalities from the initial diagnostic brain scan to the suspected recurrence, and histologically all cases were characterized by extensive gliosis; with both macrophages and reactive astrocytes present throughout. The cause of gliosis was identified as being relating to hemostatic agents in 4 cases; in the other 4 cases, the foreign-body reaction was presumed to be caused be materials used in a craniotomy or cranioplasty. This study highlights the difficulty in radiologically diagnosing a foreign-body reaction and also identifies that such a gliotic reaction may occur as a consequence of exogenous materials used in a craniotomy or cranioplasty.

## Introduction 

During neurosurgery, hemostasis is often achieved through the application of topical agents to the surgical bed, as other techniques, such as pressure application or cautery, have limited usage due to potential neurologic damage [1]. Hemostatic agents may be absorbable or nonabsorbable and function through induction of a locally-mediated inflammatory reaction that leads to thrombosis [2]. Whilst an inflammatory reaction mediated by monocytes and multinucleated giant cells is desirable initially, an exaggerated response may occur, after months or even up to decades later, resulting in concern that a resected brain tumor has recurred [3]. 

The variable proportions of acute and chronic inflammatory cells that result may lead to a ring-enhancing lesion on MRI, thereby mimicking a tumor [4]. The nature of the radiological abnormality may relate to the specific hemostatic agent used. For instance, Surgicel^®^ (Johnson & Johnson, New Jersey, USA) may result in a T1-weighted hyperintense rim along the cavity [5], or the gelatin granules contained in FloSeal^®^ (Baxter, Deerfield, USA) may result in stacked layers on T2-weighted imaging [6]. Quantitative MRI may also provide some additional information in cases of diagnostic uncertainty, such as spectroscopy; showing absent metabolites or diffusion-weighted imaging [7]. 

A number of prior case reports contain radiological and clinical information on such foreign-body reactions to hemostatic materials [8, 9, 10, 11]. However, in the case reports (or series) published to date, there are limited data available on the initial diagnostic and postoperative radiological abnormalities prior to detection of the suspected tumor recurrence. Furthermore, we are not aware of any similar reports in the literature that relate to materials used in a craniotomy or cranioplasty. Herein, we provide a series of eight cases of brain tumors with details of the radiology at three time points as well as pathological details of the tumor and gliotic reaction. 

## Materials and methods 

This study is a retrospective review of all histopathologically proven cases of a gliotic or granulomatous reaction to: (i) a hemostatic agent or (ii) materials used during craniotomy or cranioplasty. All cases were identified from a single clinical neuropathology database, we included any cases from 2009 to 2017; we chose 2009 to begin the search as MR imaging was available at three Tesla field strength from that date, thus facilitating a comprehensive review of all patient imaging at high field strength. Informed written consent was obtained at the time of the neurosurgical procedure from each patient, in line with routine clinical practice at our center. Patients in our center have routine radiological follow-up to identify tumor recurrence or progression; the frequency at which scans occur vary depending on the extent of tumor resected and the grade, thus the scanning interval in our study varied accordingly. 

Study inclusion criteria – following confirmation of a gliotic reaction histologically – were availability of: (i) initial diagnostic brain scan, (ii) histology at the time of tumor resection or biopsy, (iii) a postoperative brain scan, (iv) scan demonstrating radiological suspicion of recurrence, (v) the absence of tumor in histology from second surgery. Exclusion criteria included: (i) pathological evidence of recurrent tumor at the time of repeat excision or biopsy, (ii) cases that did not have imaging at all three time points. 

### Clinical data 

We reviewed the medical records of each patient to identify the demographic details as well as details of the surgical procedure, including the type of hemostatic agent used (if any) and also any prostheses used for cranioplasty or craniotomy. Administration of radiotherapy, following initial tumor resection, was also noted, as this could be a contributory factor to an identified gliotic reaction. 

### MRI 

All MR images were reviewed by a single experienced neuroradiologist (SL). We recorded abnormalities identified on each scan using a standardized pro forma for all cases identified as follows: (i) Diagnostic brain scan: size of T2-weighted lesion (T2WL), enhancement with gadolinium (Gd) on T1-weighted imaging [12], minimal apparent diffusion coefficient (ADC) of the solid component of the tumor [13], presence of calcification. The ADC value was recorded by placing an elliptical region of interest (ROI) within the solid component of the tumor, which was identified on the T2- and T1-weighted imaging; as all images were co-registered, this facilitated correct placement of the ROI on the ADC map. (ii) Postoperative brain scan: estimated percentage resection of tumor, diffusion-weighted imaging (DWI) abnormalities surrounding the resection cavity, Gd enhancement at resection margins. (iii) Scan with radiological suspicion of tumor recurrence: size of T2WL, Gd-enhancement, minimal ADC value of solid component of lesion. We used a t-test to compare the volumes of the T2WL, and ADC values on the diagnostic scan vs. the scan suggesting radiological recurrence, using R statistical package version 3.4.1. 

### Histopathology 

We reviewed the pathology of each tumor at the time of initial resection or biopsy and documented the subtype and WHO grade. Following confirmation of the initial neoplasm, we then reviewed the histology from the subsequent surgery, where the MRI suggested the tumor had recurred. In cases where a glioma was identified with an IDH mutation at the first surgery, sequencing was not performed following the second surgery; as there was an extremely low clinical suspicion for tumor recurrence, particularly given the florid gliotic reaction seen in each case. Immunohistochemical staining was performed: CD68 was used to confirm the presence of macrophages, and both p53 and MIB-1 were used in each case. 

## Results 

### Clinical data 

In total, we identified 8 cases fulfilling the inclusion criteria for this study. There were 4 men and 4 women, with a mean age of 39.1 ± 12.5 years. The demographics of the patients in this cohort are summarized in Table 1. Four gliomas were located in the frontal lobe, 2 in the temporal lobes, 1 thalamic (biopsy only) and metastatic lung carcinoma in the occipital lobe. 

Four cases had SurgiFlo™ used as the hemostatic agent, the other 4 had diathermy used for hemostasis. One case had a cranioplasty performed using polyetheretherketone (PEEK), and 4 cases had MatrixNEURO SterileKit used for craniotomy, with the addition of DURAFORM™ (De Puys Synthes products, NJ, USA) in 2 of these 4 cases. 

Seven of the 8 cases had a WHO grade II glioma, 1 case of metastatic lung carcinoma was identified, this patient also had stereotactic radiosurgery (SRS), none of the other 7 patients were treated with radiotherapy. 

### MRI 

In all 8 cases, the diagnostic MRI brain scan confirmed the presence of a lesion with a definite T2WL in all cases, the mean volume of the T2WL was 37.1 cm^3^ ± 35. Three of the cases (one metastatic) demonstrated Gd enhancement, and the 7 gliomas were located in either the frontal and temporal lobes and thalamus; the metastatic lung tumor was located in the occipital lobe. The mean ADC of the tumors identified was 1.4 × 10^–3^ mm^2^/s ± 0.6. The radiological abnormalities on the diagnostic brain scan are summarized in Table 2. 

Postoperatively, a brain scan confirmed a 90% or gross total resection in 7 of the 8 cases. A thalamic glioma had a biopsy performed and was not resected. Five of the cases had DWI abnormalities around the surgical cavity, providing radiological evidence of ischemic abnormalities. Two cases did not have DWI abnormalities, and a diffusion-weighted scan was not performed postoperatively in 1 case. Two of the cases demonstrated Gd enhancement at the margins of the resection. The postoperative radiological abnormalities are summarized in Table 3. 

**Figure 1.**
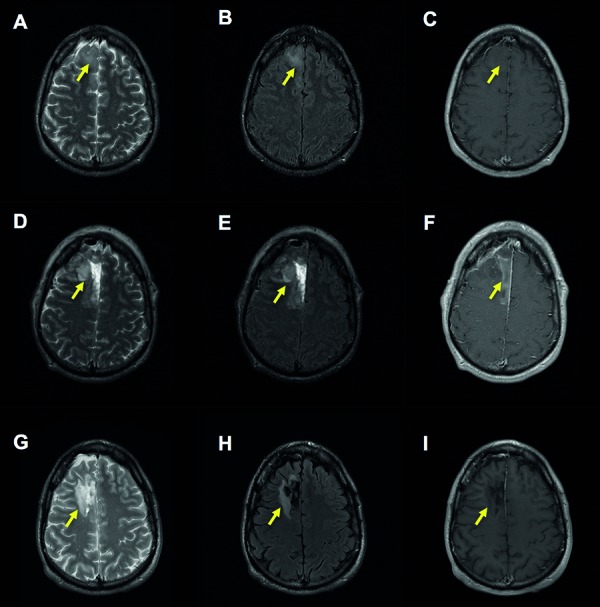
An example of MRI scans in a case with suspected tumor recurrence (case 2). Diagnostic scans (A, B, C) demonstrate a lesion in the right frontal lobe, as indicated by the yellow arrow. Postoperative images (D, E, F) were obtained within 24 hours of surgical resection of a WHO grade II oligodendroglioma (displayed in Figure 2), and display high-signal abnormality around the resection cavity on T2-weighted imaging (D and E) and hemorrhage within the cavity as a T1-weighted hyperintensity (F). Scans suggesting radiological evidence of tumor recurrence are shown (G, H), obtained 23 months from initial resection, the increase in the margin of T2-weighted abnormality surrounding the resection site, as seen in G and H, raised the suspicion of tumor recurrence. No evidence of gadolinium enhancement is seen at this time point (I). A, D, G: T2-weighted image. B, E, H: fluid attenuated inversion recovery (FLAIR) image. C, F, I: T1-weighted image post administration of gadolinium.

The mean volume of the T2WL was 35.5 cm^3^ ± 26.9. No significant differences were apparent in the T2WL volume in the diagnostic scan vs. the scan with radiologically-suspected tumor recurrence (p = 0.89). The mean ADC of the lesion identified at suspected tumor recurrence was 1.1 × 10^–3^ mm^2^/s ± 0.6. Again, no significant differences were identified between the ADC value at diagnosis vs. suspected recurrence (p = 0.87). Three scans had evidence of Gd enhancement, although this was present in discrete small nodules in each instance, rather than displaying ring-enhancement around the lesion. These radiological abnormalities are summarized in Table 4, and an example of a series of MR images is displayed in Figure 1. 

### Histopathology 

In each of the 8 cases, we reviewed the histology following the second surgery, which was performed on the basis of suspected radiological recurrence of the original tumor. 

Microscopic examination demonstrated mixed fragments of cerebral tissue, composed of cortical grey matter and subcortical white matter with overlying leptomeninges (in some but not all cases). There was focal operative acute subarachnoid and parenchymal hemorrhage. Focally, the leptomeninges showed mild chronic inflammation and were densely adherent to the cortical surface, which was partly disrupted and gliotic. Occasional mineralized neurons were seen. 

The subcortical white matter was markedly abnormal and hypercellular, showing marked oligodendrocyte hyperplasia, reactive astrocytic gliosis, and macrophage infiltration. Focally, the white matter was pale and rarefied in appearance. Significant cellular atypia was not seen nor were mitoses, necrosis, or microvascular proliferation. Eosinophilic granular bodies or Rosenthal fibers were not seen in any cases studied. 

As expected, there were several foci of perivascular lymphocytic chronic inflammation and foci of hemosiderin pigment deposition, indicative of prior hemorrhage at the surgical sites in a number of cases. At the deep aspect of the white matter in several tissue fragments, there were features of a cystic cavity relating to prior surgery, which was surrounded by thin gliovascular septations, foamy macrophages, and large reactive hypertrophied astrocytes. Occasional tissue fragments were partly lined by ependyma. 

**Figure 2.**
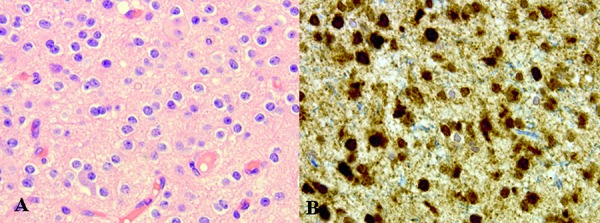
Histology of lesion identified on MRI scan in Figure 1 (A, B, C), demonstrating an oligodendroglioma WHO grade II. A: H & E × 40. B: IDH mutant × 40 – demonstrated using IDH1 R132H immunohistochemical stain.

**Figure 3.**
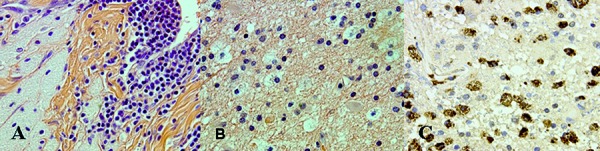
Histology of lesion identified in Figure 1 (G, H, I) suspected to be recurrence of tumor. A: Amorphous material and chronic inflammation H & E × 40. B: Macrophage accumulation × 40. C: CD68 confirms macrophage accumulation × 40.

Immunohistochemistry demonstrated dense gliosis within the subcortical white matter using GFAP. Mutant IDH1 R132H (immunohistochemical stain), performed on several tissue blocks, was not expressed in any of the 4 cases where a WHO grade II glioma with a IDH1 R132H mutation was identified at the initial surgery, an example is shown in Figure 2. CD68 highlighted the florid degree of macrophage infiltration, demonstrated in Figure 3. Significant nuclear immunopositivity for p53 was not identified in any of the specimens. The proliferation index, assessed by MIB-1, was universally low. 

No evidence of tumor recurrence was identified in any of the eight specimens, and histological abnormalities were found to be gliotic only in nature. 

## Discussion 

There are three novel findings in this study. Firstly, we evaluated the MRI data obtained at three time points, rather than solely at the suspected radiological recurrence of tumor. Secondly, we obtained quantitative MRI information, using the ADC map, on the lesions identified. Lastly, we identified pathological evidence of a gliotic reaction to both hemostatic agents and synthetic materials used in craniotomies and cranioplasties, which resulted in radiological abnormalities compatible with tumor recurrence. 

In this present study, we analyzed MRI at three time points: initial diagnosis, postoperatively, and at the time of suspected recurrence. This is in contrast to prior reports, where the radiological abnormalities due only to the foreign-body reaction are provided [4, 6, 9, 10, 14]. The data obtained in this study indicate that there were no significant differences in the volume of the T2-weighted lesion at tumor diagnosis and at the time of the gliotic changes identified. 

These findings highlight the diagnostic difficulty, based on MRI alone, that faces the neuroradiologist when an expanding abnormality is identified post-tumor resection. Pathologically increased T2-weighted signal may be seen due to demyelination, edema, and breakdown in the blood-brain barrier [15], all of which may be seen in the context of a gliotic reaction or indeed in reactive changes [4]. For this reason, other MR imaging sequences, including quantitative measures and additionally positron emission tomography (PET) have been investigated as potential discriminatory tools between tumor progression and gliosis, which may mimic recurrence [16]. 

In this study, we also obtained measurements of the ADC from the solid component of the tumor at diagnosis and from the gliotic reaction. We did not observe any significant differences in the values obtained. The ADC value is inversely correlated to the cellularity of a tumor [13], therefore, the similar ADC values due to reactive abnormalities suggest that similarly closely congested cellularity occurs in this context. We confirmed this hypothesis pathologically through our observation that the gliosis consisted primarily of densely packed macrophages as well as numerous reactive astrocytes, which would account for the reduction in ADC within the gliotic reaction. This quantitative MRI finding further highlights the difficulty in discriminating between tumor and foreign-body reaction in vivo. 

The majority of prior reports of foreign body reactions mimicking tumors relate to hemostatic agents used [9, 10, 11]. Our findings are in agreement with these prior reports, as 3 of the cases of gliotic reaction were attributed to hemostatic agents used during initial surgical resection. However, in addition, we also identified 5 patients in whom no hemostatic agents were used, and who similarly developed reactive abnormalities following a cranioplasty or craniotomy where exogenous prostheses were employed. 

In 1 such case, a PEEK cranioplasty was performed. Whilst the complication rate of a PEEK cranioplasty is estimated to be ~ 1/3 [17], to our knowledge, there are no prior reports of an abnormal reaction mimicking a tumor [18]. In the 4 other patients MatrixNEURO SterileKit was used, and in 2 patients DURAFORM™ was used additionally. We are also not aware of prior reports of such intense gliosis mimicking a tumor following the implementation of such materials surgically. 

Whilst we identified three novel findings in relation to the radiological and pathological abnormalities in this present study, a number of limitations need to be considered. Firstly, we identified all available cases in our database, the sample size of 8 restricted a more robust statistical analysis between radiological abnormalities and pathological correlates. This could be addressed in a future multicenter study, thereby facilitating inclusion of a larger cohort with an expanded dataset. 

Secondly, whilst diffusion-weighted MRI data were available, other quantitative MR measures, such as perfusion or diffusion tensor imaging were not available in this current study [16]. Perfusion data or DTI may provide additional in vivo data that could be used to discriminate between a gliotic reaction and tumor progression. A future study with a more comprehensive MRI protocol, containing both conventional as well as quantitative sequences, could provide such data. 

Thirdly, we did not have sufficient clinical information available on review of the patient’s medical records to identify any possible risk factors to predispose the patients to a foreign-body reaction. A future study containing information on atopy, asthma, or a history of prior allergies or HLA typing may lead to identification of particular risk factors that predispose patients to a gliotic reaction following exposure to exogenous surgical materials. 

In conclusion, we identified three novel findings. Firstly, comparison of the diagnostic scan vs. the scan with suspected tumor recurrence may not obfuscate the need for surgical intervention due to diagnostic difficulty in discriminating between a gliotic reaction and tumor recurrence in vivo. Secondly, an abnormality in diffusion-weighted signal may be observed in reactive changes, analogous to that seen in a glioma, further emphasizing the difficulty in radiologically diagnosing a gliosis. Finally, whilst reactive gliosis has been well documented in response to hemostatic agents, this study highlights that identical pathological abnormalities may also be observed in response to exogenous materials used in a craniotomy or cranioplasty. 

## Funding 

No funding source was obtained to perform this study. 

## Conflict of interest 

The authors declare no conflict of interest. 

**Table 1.** Demographics of patients identified who had a gliotic reaction to a surgical hemostatic agent during surgical procedure. The mean age is 39.1 ± 12.5 years, and mean interval prior to suspected radiological recurrence was 28.1 ± 23.9 months. All cases had an initial resective procedure apart from the thalamic lesion that was biopsied only.

Case number	Age (years)	Gender	Site of resection/biopsy	Hemostatic agent used/prostheses inserted for craniotomy	Histology from first surgical resection/biopsy	Interval from surgery to radiological suspicion of tumor recurrence (months)
1	28	M	Right frontal	SurgiFlo™/None	Astrocytoma WHO II	49
2	40	M	Right frontal	Diathermy/Polyetheretherketone (PEEK) cranioplasty	Oligodendroglioma WHO II	23
3	39	F	Left frontal	Diathermy/MatrixNEURO SterileKit	Oligodendroglioma WHO II	13
4	23	M	Right temporal	SurgiFlo™/None	Astrocytoma WHO II	9
5	33	F	Right temporal	Diathermy/MatrixNEURO SterileKit and DURAFORM™	Oligodendroglioma WHO II	19
6	64	M	Occipital	Diathermy/MatrixNEURO SterileKit	Metastatic lung carcinoma	14
7	46	F	Left frontal	SurgiFlo™/None	Oligodendroglioma WHO II	19
8	40	F	Right thalamus	SurgiFlo™/MatrixNEURO SterileKit and DURAFORM™	Astrocytoma WHO II	79

**Table 2.** Summary of radiological abnormalities recorded on diagnostic brain scan when the tumor was first identified radiologically prior to neurosurgical intervention.

Radiological abnormalities	Case number							
	1	2	3	4	5	6*	7	8
Size of T2-weighted lesion (cm)	4 × 1.5 × 2	6 × 3 4.5	5 × 4.5 × 4	3 × 2 × 2	3.5 × 2.5 × 2	2 × 2 × 2	4 × 4 × 4	3 × 2 × 2
Enhancement with Gd	No	No	No	No	No	Yes	Yes	Yes
Minimum ADC value of solid component (× 10^–3^ mm^2^/s)	N/A	N/A	1.4	2.1	1.1	0.5	1.3	N/A
Location – lobe	Frontal	Frontal	Frontal	Temporal	Temporal	Occipital	Frontal	Thalamus
Calcification	No	No	No	No	No	No	No	No

*Metastatic lung cancer – all other tumors are WHO grade II gliomas. Gd = gadolinium; ADC = apparent diffusion coefficient.

**Table 3.** Summary of radiological abnormalities recorded on postoperative brain scan.

Radiological abnormalities	Case number							
	1	2	3	4	5	6	7	8
Estimated % resection of tumor	90%	GTR	90%	GTR	GTR	GTR	GTR	Biopsy – no resection
DWI abnormalities surround the lesion (cm)	5	1.5	Small patchy foci	Small patchy foci	None	None	2	N/A
Enhancement with Gd at margins	No	No	No	No	No	No	Yes	Yes

Gd = gadolinium; GTR = gross total resection – identified radiologically; DWI = diffusion weighted imaging.

**Table 4.** Summary of radiological abnormalities recorded on scan, with suspected recurrence of tumor radiologically.

Radiological abnormalities	Case number							
	1	2	3	4	5	6	7	8
Size of T2-weighted lesion (cm)	5 × 2.5 × 3	4.5 × 1.5 × 2	5 × 4.5 × 4	4 × 2.3 × 3	6 × 3 × 3	1.3 × 1.6 × 1	4 × 2 × 4	3 × 3 × 3
Enhancement with Gd (yes or no)	No	No	Yes – enhancing nodule	No	No	Yes	Yes – 1 cm enhancing nodule	Yes – three 1 cm enhancing nodules
Minimum ADC value of solid component (× 10^–3^ mm^2^/s)	1.3	1.5	N/A	1.8	0.1	1.5	0.8	0.8

Gd = gadolinium; ADC = apparent diffusion coefficient.
